# Trajectories and predictive significance of inflammatory parameters for clinical outcome in COVID–19 patients treated with tocilizumab

**DOI:** 10.1007/s15010-024-02375-x

**Published:** 2024-08-29

**Authors:** Alexander Killer, Smaranda Gliga, Pascal Massion, Carla Ackermann, Clara De Angelis, Charlotte Flasshove, Noemi Freise, Nadine Lübke, Jörg Timm, Kirsten Alexandra Eberhardt, Johannes Bode, Björn-Erik Ole Jensen, Tom Luedde, Hans Martin Orth, Torsten Feldt

**Affiliations:** 1 Department of Gastroenterology, Hepatology and Infectious Diseases, Medical Faculty and University Hospital Düsseldorf, Heinrich Heine University, Moorenstraße 5, 40225 Düsseldorf, Germany; 2 Institute of Virology, Medical Faculty and University Hospital Düsseldorf, Heinrich Heine University, Düsseldorf, Germany; 3 Department of Tropical Medicine, Bernhard Nocht Institute for Tropical Medicine and I. Dep. of Medicine, University Medical Center Hamburg-Eppendorf, Hamburg, Germany

**Keywords:** COVID-19, Tocilizumab, ARDS, IL-6, Hyperinflammation, CRP

## Abstract

**Purpose:**

The IL-6 receptor inhibitor tocilizumab reduces mortality and morbidity in severe cases of COVID-19 through its effects on hyperinflammation and was approved as adjuvant therapy. Since tocilizumab changes the levels of inflammatory markers, we aimed to describe these changes in patients treated with tocilizumab, analyse their value in predicting death and bacterial superinfection and determine their influence on mortality rates.

**Methods:**

A retrospective analysis of 76 patients who were treated with tocilizumab for severe COVID-19 in 2020 and 2021 was conducted. Inflammatory markers (IL-6, C-reactive protein (CRP), procalcitonin) were documented before and up to seven days after tocilizumab administration.

**Results:**

The overall mortality was 25% and 53.8% in patients who required invasive respiratory support. Deceased patients had higher baseline IL-6 (p = 0.026) and peak IL-6 levels after tocilizumab vs those who survived (p < 0.0001). A peak IL-6 value > 1000 pg/dl after tocilizumab administration was a good predictor of mortality (AUC = 0.812). Of the deceased patients 41.1% had a renewed CRP increase after an initial decrease following tocilizumab administration, compared to 7.1% of the surviving patients (p = 0.0011). Documented bacterial superinfections were observed in 35.5% (27/76) of patients, of whom 48.1% (13/27) died.

**Conclusion:**

CRP-decline and IL-6 increase after tocilizumab treatment occurs regularly. An increase of IL-6 levels exceeding tenfold of baseline IL-6 levels, an absolute peak of 1000 pg/ml or a renewed increase of CRP are associated with higher mortality. Suppressed CRP synthesis can impede the diagnosis of bacterial superinfections, thus increasing the risk for complications.

**Supplementary Information:**

The online version contains supplementary material available at 10.1007/s15010-024-02375-x.

## Introduction

SARS-CoV-2 infection can lead to pneumonia and in severe cases to acute respiratory distress syndrome (ARDS) which is often associated to a hyperinflammatory condition which is also referred to as “cytokine storm” [1]. Since the first cases were reported, treatment was focused on antiviral concepts [2]. After early clinical studies using drugs like lopinavir or hydroxychloroquine, intended to have an antiviral effect, were not showing beneficial effects on the course of COVID-19, remdesivir showed positive results in preventing severe disease [3] and is still established as the treatment of choice for patients with early disease or patients with COVID-19 pneumonia requiring oxygen supplementation. Later during the pandemic, several monoclonal antibodies [4, 5] and other antiviral treatments such as nirmatrelvir/ritonavir followed [6]. For patients in later stages of COVID-19 and with a missed window of opportunity to prevent pneumonia, i.e. patients on supplemental oxygen or any form of ventilatory support, the RECOVERY trial showed a benefit of dexamethasone treatment [7]. Following this concept of anti-inflammatory treatment, other anti-inflammatory drugs have been tested [2]. Because of its method of action and being an established drug for rheumatoid arthritis [8], the IL-6 receptor blocker tocilizumab came into focus for treating hyperinflammation [9]. Outcomes for tocilizumab varied in different trials, most likely due to different timing of tocilizumab use and different cohorts, leading to discussions regarding its efficacy [10–13]. Several guidelines, including the living WHO guideline recommend tocilizumab for patients with severe COVID-19 and acute worsening of the disease [14, 15].

In the clinical management of these patients, there remains the problem of differentiating possible bacterial superinfection and COVID-19 related hyperinflammation, especially in cases of acute respiratory distress syndrome (ARDS) and intensive care treatment. Since tocilizumab blocks membrane-bound and soluble IL-6 receptors, its application is followed by an increase in free serum IL-6 levels. This is mediated by the inhibition of its IL-6R–mediated binding and consumption due to the unavailability of tocilizumab-free IL-6R. The increased level of free IL-6 during tocilizumab treatment most likely reflects actual endogenous IL-6 production and true inflammatory activity [16]. A decrease of C-reactive protein (CRP) was observed as a secondary effect of the inhibition of IL-6 signaling.

Since bacterial infection is marked by an increase in IL-6 and CRP levels, the usefulness of these markers after tocilizumab treatment is unclear. In addition, whether these markers are still applicable for the evaluation of disease progression in regarding COVID-19, and which thresholds may be used for interpretation, is a question of interest. Therefore, we analyzed the kinetics of inflammatory markers and their predictive value for unfavorable clinical outcomes and bacterial superinfections in tocilizumab treated COVID-19 patients. We also want to guide clinicians in the interpretation of inflammatory markers after tocilizumab therapy: What changes can be expected and therefore regarded as usual/normal?

## Materials and methods

In this retrospective, single centre cohort study we included 76 patients diagnosed with SARS-CoV-2 infection (defined as detection of SARS-CoV-2 RNA from naso-pharyngeal secretions) who were admitted to the infectious diseases unit or intensive care unit (ICU) at the University Hospital in Düsseldorf between February 2020 and 31st of December 2021 and received tocilizumab therapy. For each patient, clinical parameters and inflammation markers (IL-6, CRP and procalcitonin (PCT)) were collected before (baseline) and up to a week after tocilizumab administration.

The main objectives were to describe changes in levels of inflammatory markers and analyse their value in outcome prediction, bacterial superinfection and determine the influence on mortality rates. As a secondary objective, we analysed pattern of tocilizumab use and in-hospital mortality and morbidity.

### SARS-CoV-2 stages of disease

Disease stage definitions were retrieved from the Lean European Open Survey on SARS-CoV-2-infected Patients (LEOSS) cohort: uncomplicated, complicated, and critical illness. The characteristics of each phase have been previously described [17].

### Confirmation of SARS-CoV-2 infection

#### Isolation of viral genomic material and SARS-CoV-2 quantification

SARS-CoV-2 was detected in respiratory secretions collected with nasopharyngeal swabs using the cobas® SARS-CoV-2 test on the cobas®6800 system (c6800, Roche Diagnostics) or the SARS-CoV-2 test on the NeuMoDx™ platform (NDX, Qiagen) as described previously by Freise et al. [18].

### Tocilizumab administration

In the majority of patients, tocilizumab was administered as a single-shot, weight-adapted (8 mg/kg bodyweight) intravenous infusion. In individual cases, a second or third dose was administered after careful consideration. For this analysis we only included first tocilizumab administration.

Tocilizumab was administered during progressive respiratory deterioration, typically in the first 24 h after a rapid increase in oxygen demand to 6–8 l/min or after admission to the ICU for initiation of high-flow O2 administration or non-invasive or invasive ventilation. Before tocilizumab administration the likelihood of bacterial superinfection was assessed by clinical evaluation, CT scan and evaluation of laboratory results. Until the approval by the European Medicines Agency (EMA) in December 2021 patients signed an informed consent information for off-label use. After approval, tocilizumab was used within the scope of the approval and national therapy recommendations.

### Statistical analysis

Data were analysed using GraphPad Prism Version 9.5.1 (Boston, MA, USA). Simple frequencies and descriptive analyses were performed. A mixed-effects model was used to test association between time and inflammatory marker values. A non-parametric Mann–Whitney test was used to determine differences in outcomes between the various subgroups. Differences were considered statistically significant at a two-tailed p < 0.05.

Receiver operating characteristic (ROC) curve analysis was used to establish inflammatory marker (IL-6, CRP) cut-offs that predict mortality. Sensitivities and specificities were obtained for every possible IL-6 and CRP cut-off.

For illustration of a directed acyclic graph (DAG) we used the statistical software R (R Core Team 4.3.0. R: A Language and Environment for Statistical Computing. R Foundation for Statistical Computing, Vienna, Austria).

### Ethical considerations

The ethical committee of the Heinrich-Heine-University Düsseldorf approved the retrospective analysis of the cohort (Study number: 2021–1385/Amendment 2021-1385_1).

### Data availability

The datasets generated and analysed are available from the corresponding author on reasonable request.

## Results

Between February 2020 and December 2021, 76 predominantly male patients (72.4%) were included in this retrospective analysis (Table 1). Patients had symptoms for a median of 6 days before COVID-19 diagnosis (interquartile range (IQR) 3–7 days).

**Table 1:** Patient characteristics

Variable	n	Mortality (n/%)
Total	76	19 (25%)
Age		
< 35 years	5	0 (0)
36–65 years	46	6 (13)
66–85 years	23	12 (52.2)
> 85 years	2	1 (50)
Gender		
Male	55	15 (27.3)
Female	21	4 (19)
BMI		
18.5–24.9 kg/m2	5	0 (0)
25–29.9 kg/m2	17	6 (35.3)
30–34.9 kg/m2	15	5 (33.3)
> 34.9 kg/m2	14	2 (14.3)
Unknown	25	6 (24)
Stage of disease at diagnosis		
Uncomplicated phase	9	3 (33.3)
Complicated phase	39	6 (15.4)
Critical illness	28	10 (35.7)
Stage of disease at tocilizumab administration		
Complicated phase	26	4 (15.4)
Critical illness	50	15 (30)
Invasive/non-invasive/ECMO at tocilizumab administration		
NIV	24	5 (20.8)
IV	9	5 (55.6)
ECMO	4	2 (50)
New invasive/non-invasive/ECMO after tocilizumab administration		
NIV	20	2 (10)
IV	10	4 (40)
ECMO	10	7 (70)
No change/improved	33	5 (15.2)
Unknown	3	1 (33.3)
Duration ECMO (median, range)	7 (1–36) days	
Duration of hospital stay for surviving patients (n = 57)		
≥ 14 days	34	
< 14 days	23	
Duration invasive/non-invasive ventilation		
< 3 days	4	0 (0)
3–15 days	42	10 (23.8)
> 15 days	14	7 (50)
None	15	2 (13.3)
Unknown	1	0 (0)
Comorbidities		
Malignancies (solid tumor)	2	1 (50)
Solid organ transplantation	1	1 (100)
Diabetes mellitus	21	6 (28.6)
Hypertension	34	12 (35.3)
Dialysis	2	2 (100)
Thrombo-embolic complications (DVT, PE, abdominal thrombosis)	3	1 (33.3)
Other therapies		
Hydroxychloroquine	6	2 (33.3)
Lopinavir/ritonavir	2	1 (50)
Remdesivir	27	7 (25.9)
Dexamethasone	59	12 (20.3)
Prednisolone (high dose)	3	2 (66.7)
Casirivimab/Imdevimab	6	0 (0)

*n* number, *ECMO* extracorporeal membrane oxygenation, *NIV* non-invasive ventilation, *IV* invasive ventilation, *DVT* deep venous thrombosis, *PE* pulmonary embolism

Most patients had one or more cardiovascular risk factors (age > 65 years, male sex, obesity, diabetes, hypertension), and only three were severely immunocompromised (solid organ transplantation, active malignancy). At the time of COVID-19 diagnosis, nine patients were in the uncomplicated phase of COVID-19 and did not require oxygen supplementation. At the time of tocilizumab administration, all nine of them had progressed to either a complicated or critical disease stage. Specifically, five patients required oxygen supplementation, two required non-invasive ventilation (NIV) and one required invasive ventilation. Of the 39 patients in the complicated stage at COVID-19 diagnosis, 10 required non-invasive ventilation at the time of tocilizumab administration and one was intubated. Twenty-eight patients were already critically ill at COVID-19 diagnosis.

The total in-hospital mortality rate was 25% but increased to 53.8% in the subgroup of patients who required invasive ventilation or extracorporeal membrane oxygenation (ECMO) at the time of tocilizumab administration.

Fourteen patients, mostly treated at the beginning of the pandemic, did not receive concomitant dexamethasone therapy (n = 59) or high-dose prednisolone (n = 3). The mortality rate in this group was 35.7% vs. 20.3% (hazard ratio (HR) 1.8) in the subgroup of patients who received dexamethasone (p = 0.29). There was no statistical difference in mortality between patients who had already received dexamethasone (31/59) at the time of tocilizumab administration and those who had received it simultaneously with tocilizumab (28/59) (22.6% vs. 14.3%, p = 0.51).

### Antibiotic therapy and bacterial superinfection

A total of 48 patients received antibiotic therapy. The mortality rate in this subgroup was 31.3% vs. 14.3% in patients who did not receive antibiotic therapy (p = 0.17). Antibiotic therapy was often administered because of high levels of inflammatory markers (CRP, PCT), but only 27/48 patients had evidence of a bacterial superinfection, either through positive microbiological samples or based on diagnostic imaging, as detailed in Table 2. The rate of bacterial superinfection was 35.5%, with mortality in this group being 48.1% (13/27), compared to 12.2% (6/49) in patients without bacterial superinfection (p = 0.0009). This reflects the severity of bacterial superinfection regarding the clinical outcome.

**Table 2:** Bacterial superinfections in patients who received tocilizumab

Nr.Pat	Diagnosis	Positive microbiological sample					
		Blood culture	Urine	Tracheal/bronchial secretion	Catheter culture	Screening (nose-throat, rectal swab)	Diagnostic imaging
3	Septic shock, HAP	S. pneumoniae		S. pneumoniae			progressive lung infiltrates
6	HAP						progressive lung infiltrates
7	Septic shock	cogulase-negative Staphylococci					
8	Septic shock	Cutibacterium					
9	Sepsis	S. maltophilia, Cutibacterium, E. faecium		Strenotrophomonas maltophilia	E. faecalis, E. faecium		
10	Septic shock, intestinal ischaemia					VRE	
11	Septic shock	cogulase-negative Staphylococci, Cutibacterium					
13	HAP						progressive lung infiltrates
16	Sepsis, HAP	MSSE					
17	Sepsis, HAP	MRSE		Candida alb			
18	Sepsis, HAP	E.faecium, Staph. epidermidis, Candida glabrata		Candida glabrata, Serratia marscenses		E. faecium	
19	Cholecystitis, liver abscess, HAP						cholecystitis, progressive infiltrates
21	Sepsis	MSSA					
34	HAP			MSSA, Proteus, Klebsiella oxytoca			
35	HAP			Candida alb			
40	HAP		Klebsiella pneumoniae	Klebsiella pneumoniae		cogulase-negative Staph	new infiltrates, pleural effusions, pericardial effusion
42	HAP			Serratia liquefaciens			
52	Sepsis	Fusobacterium					
53	Septic shock	MRSE		Candida tropicalis, Serratis marcescens			
55	Pleural empyema, sepsis	Staphylococcus capitis		Staphylococcus hominis	MSSE		large pneumatocele, pleural effusion
57	Sepsis	Staphylococcus hominis		Enterobacter cloacae			
58	Sepsis	MRSE, MSSA					
59	Septic shock	MSSA					
61	Sepsis, HAP	Streptococcus mitis, MSSE		MSSA, Haemophilus influenzae			progressive Infiltrates
63	Sepsis	Staphylococcus hominis, Staphylococcus capitis					
70	Endocarditis						endocarditis
73	Sepsis	Candida glabrata					

*HAP* Hospital-acquired pneumonia, *VRE* vancomycin-resistant enterococci, *MSSA/MRSA* methicillin-sensitive/resistant Staphylococcus aureus, *MSSE/MRSE* methicillin-sensitive/resistant Staphylococcus epidermidis

### Evolution of inflammatory markers

**Fig. 1:**
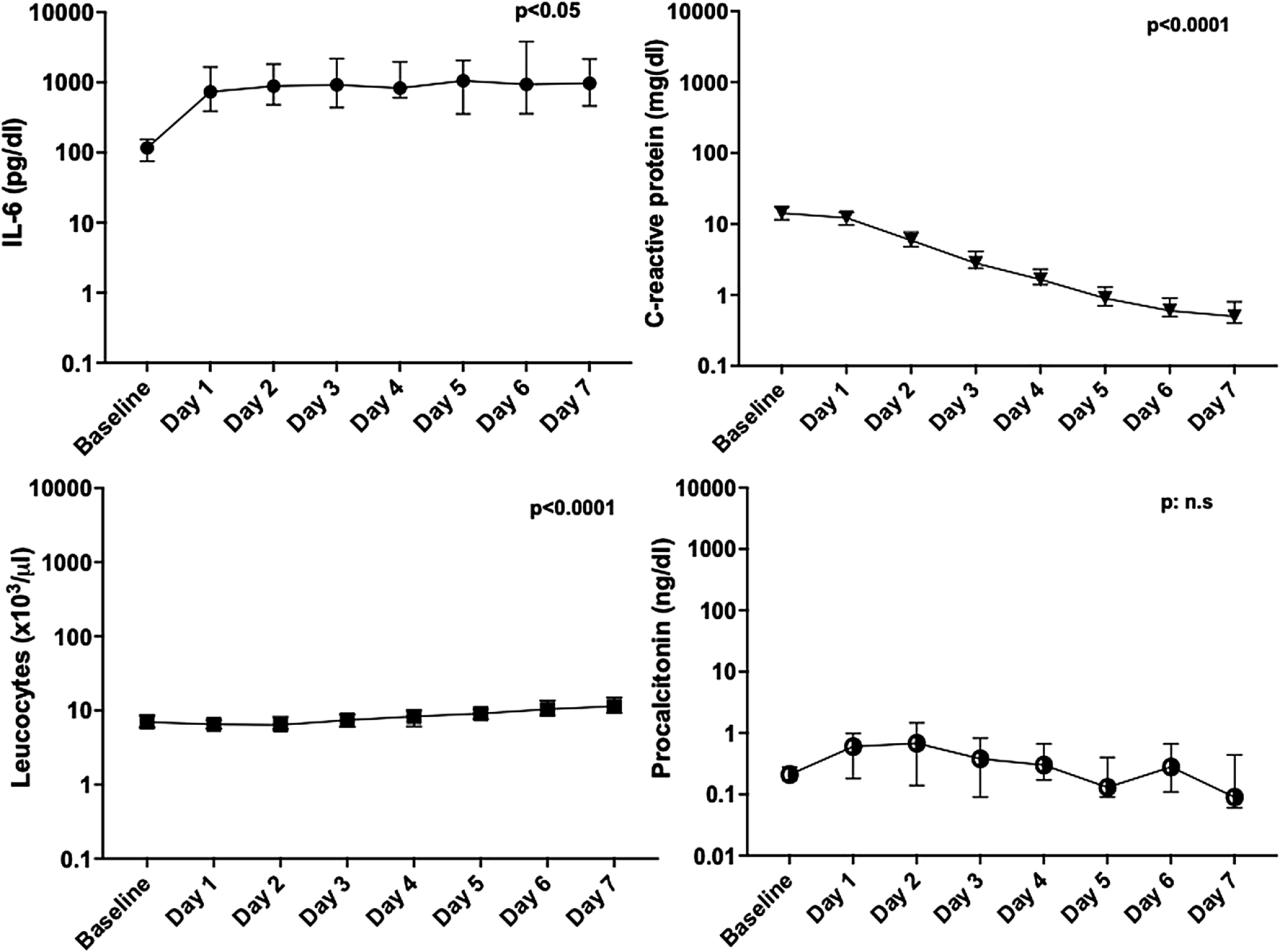
Evolution of inflammation markers from baseline (before tocilizumab) up to day seven after tocilizumab administration. Medians with 95% confidence intervals and p-values of the mixed effects analysis are depicted. The Y-axis is in a logarithmic scale

**Fig. 2:**
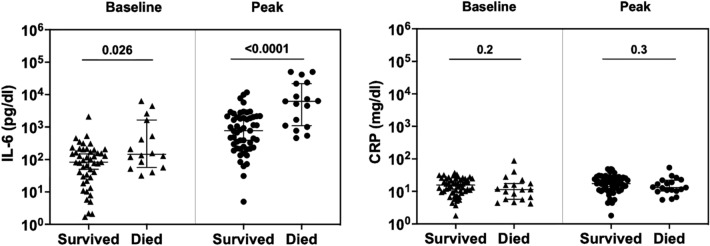
Differences in baseline and peak values of inflammatory markers (IL-6, CRP) between survived and deceased patients. Medians with 95% confidence intervals and p-values are depicted. The Y-axis is in a logarithmic scale using a power of 10 representation

At baseline, the median (IQR) values for inflammatory markers were as follows: IL-6: 116.5 (40.4–209) pg/dl, CRP: 14.3 (9.2–22.7) mg/dl PCT: 0.21 (0.11–0.69) ng/dl. After tocilizumab administration, the median levels of IL-6 increased (approximately by 10 times at day 1–2) and the median levels of CRP progressively declined (approximately by 10 times at day 7), as detailed in Fig. 1. However, any new CRP increase after initial decline was observed for 52.6% (10/19) of the patients who died vs. 14% (8/57) of those who survived (p = 0.0014). Furthermore, the IL-6 median values before (145.2 vs. 83 pg/dl, p = 0.026) and after Tocilizumab administration (6224 vs. 770 pg/dl, p < 0.0001) (Fig. 2), as well as the increase of IL-6 levels after administration of tocilizumab (23.7-fold (confidence interval (CI) 95% 9.5–243.8) vs 10.5-fold (CI 95% 3.1–32.1), were higher in the subgroup of patients who died compared to those who survived.

In a ROC curve analysis, an IL-6 factor increase of 10.7 had a sensitivity of 75% and a specificity of 56.1% in predicting mortality but the test only had a moderate predictive value (area under curve (AUC) = 0.675, CI 95% 0.52–0.83). A better predictive value (AUC = 0.812, CI 95% 0.694–0.929, sensitivity 82.4%, specificity 56.6%) was shown for a peak IL-6 value higher than 1000 pg/dl after tocilizumab administration.

**Fig. 3:**
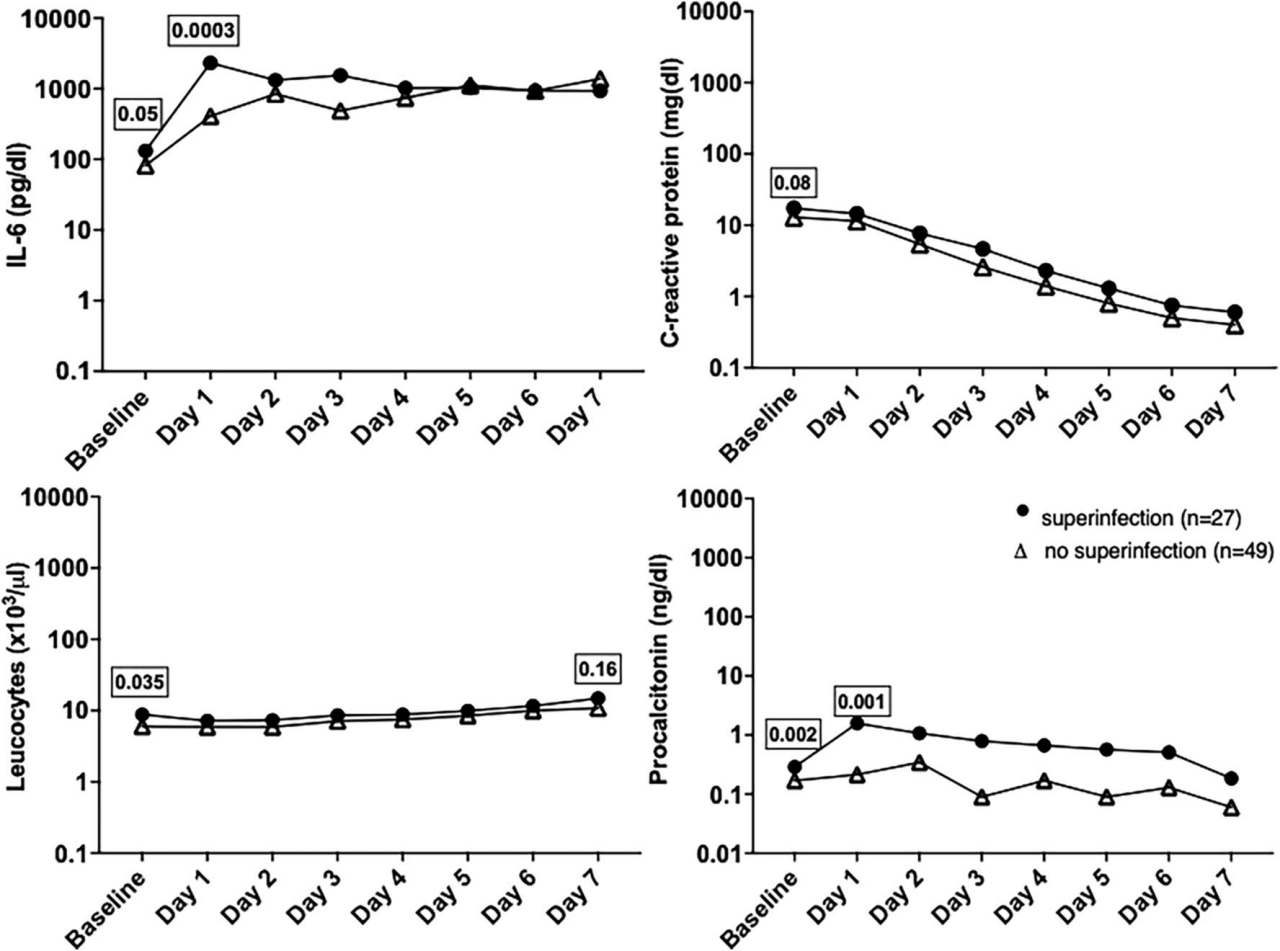
Evolution of inflammatory markers in the subgroups of patients with superinfection (n = 27, full point) and those without superinfection (49, empty triangle) from baseline up to seven days after tocilizumab administration. Only median values and the p-values of the Mann–Whitney test for baseline/peak values are depicted on the graphic. The Y axis is in a logarithmic scale

In the subgroup of patients with microbiological evidence or suggestive imaging of bacterial infection (27/76), the median baseline (131.7 pg/dl vs 81.9 pg/dl) and peak IL-6 (2155 pg/dl vs. 425.5 pg/dl) values were higher than in patients without superinfection (p = 0.05 and p = 0.0003 respectively). Furthermore, the median PCT values at baseline were significantly higher in the group of patients with superinfection (0.29 vs 0.17 ng/dl, p = 0.002) and remained higher throughout the follow-up period vs patients without superinfection. Figure 3 shows that patients with superinfection also had higher baseline leucocyte counts but there were no differences in the subgroups in the follow-up period. The same can be said for the CRP values.

The influence of peak IL-6 after tocilizumab administration on mortality (odds ratio [OR] 4.11 for IL-6 > 1000 pg/dl post-tocilizumab, 95% CI 0.96–17.7), as well as potential confounding factors are illustrated in Supplementary Fig. 1.

## Discussion

### Role of glucocorticoid therapy

Our data support the use of dexamethasone therapy for patients with severe COVID-19 by demonstrating an HR for death of 1.8 for patients who did not receive dexamethasone after tocilizumab use. Therefore, tocilizumab should be combined with dexamethasone. The fact that some patients were only treated with tocilizumab is due to the early stage of the pandemic and the lack of data that followed the RECOVERY study, leading to EMA approval of tocilizumab for COVID-19 patients with dexamethasone treatment and clinical worsening, therefore tocilizumab use without prior dexamethasone treatment would now be considered off-label and is not recommended. Of note, there was no difference between patients whose dexamethasone treatment had been started before tocilizumab administration and those whose dexamethasone treatment was started simultaneously with tocilizumab administration. Therefore, the downregulation of immune response with tocilizumab alone appears insufficient, while the timing of tocilizumab administration in the phase of clinical deterioration, independent of prior dexamethasone treatment, seems to be of utmost importance. As previously discussed by Hasanin and Mostafa [11], studies with a high proportion of patients receiving glucocorticoids and tocilizumab simultaneously, such as REMAP-CAP [19] and COVINTOC [20], showed better outcomes than studies with high proportion of tocilizumab monotherapy [21, 22].

### Interpretation of inflammation markers after tocilizumab treatment and role of IL-6 in assessment of COVID-19

According to its method of action of blocking the IL-6 receptor, we report an overall increase in IL-6 after tocilizumab treatment to a tenfold compared to baseline IL-6 values for all patients, regardless of outcome. This means that, in general, an increase of IL-6 after tocilizumab treatment should not be considered to be a sign of clinical worsening and should not provide reason for further anti-infective treatment for suspected bacterial superinfection without supporting clinical features. However, we report that in the subgroup of surviving patients IL-6 levels increased less markedly, (770 pg/dl in median), than in non-surviving patients (6224 pg/dl in median). In addition, higher baseline IL-6 values, linked to higher overall inflammation and therefore severity of disease, are related to higher mortality (median non-survivors 145.2 vs. survivors 83 pg/dl). Baseline IL-6 levels are known to be associated with clinical outcome [23]. The differences in IL-6 levels between non-survivors and survivors are even more intense after tocilizumab treatment (eightfold after tocilizumab vs. 1.7fold at baseline). This may be interpreted as revelation of the actual production of IL-6 by blocking its absorption [16]. Another explanation could be the interruption of the IL-6 feedback loops [24].

Both findings underline the role of IL-6 in the assessment of severe COVID-19. A significant increase of IL-6 should trigger the evaluation of intensifying the anti-inflammatory treatment by e.g. tocilizumab use. Patients with high IL-6 baseline levels not treated with tocilizumab and patients with low IL-6 at baseline treated with tocilizumab reportedly have higher mortality rates [25]. This shows that COVID-19 treatment needs to be individualized and especially patients with high IL-6 may benefit from tocilizumab treatment. IL-6 as a predictive marker for COVID-19 is well established [26, 27], it is also reportedly predictive of mortality in patients with cardiovascular disease [28] or end stage kidney disease [29].

We also reported that patients with clinical constellation of bacterial superinfection and microbiological confirmation had CRP levels similar to those without superinfection, but in contrast, IL-6 levels were higher in patients with superinfection. Therefore, after tocilizumab treatment IL-6 may be used to evaluate infectious complications, whereas CRP remains less meaningful. Relying on CRP as a sole inflammatory marker has been shown to lead to severe infections being overlooked in patients with rheumatoid arthritis under tocilizumab treatment [30]. The effect of tocilizumab on CRP levels is reported to last for about 14 days, while white blood cell count and procalcitonin are less affected [31].

### Using changes of inflammatory markers after tocilizumab treatment for outcome prediction

An IL-6 increase after tocilizumab treatment by factor 10.7 had a sensitivity of 75% and specificity of 56.1% for predicting mortality, but an IL-6 peak of > 1000 was a better predictor. This is mostly due to the fact that patients in our cohort who received tocilizumab at a late stage of disease with already very high IL-6 levels. The predictive value of IL-6 increase should be re-evaluated for a larger cohort including only patients receiving tocilizumab before initiating invasive ventilation. A CRP increase after tocilizumab treatment was also predictive of mortality, while we did not observe increased CRP levels in patients with microbiological evidence of bacterial superinfection, meaning rising CRP levels may be rather reflecting progression of COVID-19 in this situation.

### Evaluation of inflammatory markers after tocilizumab administration

IL-6: As previously stated IL-6 levels rise after administration but still allow identification of patients at risk, Therefore IL-6 can and should be used to monitor patients with severe COVID-19 disease.

CRP: CRP levels decrease after treatment and do not reveal bacterial superinfection, leading to a diagnostic blind spot. CRP increase is still associated with negative outcome.

PCT: Procalcitonin is the least influenced marker in our analysis and was associated with identification of bacterial superinfection. Therefore, PCT could still be used for that cause.

Leucocyte count: After tocilizumab administration there was no difference between patients with or without superinfection. This lack of diagnostic value could also be due to regular increase of leucocyte count following dexamethasone treatment, underlining the diagnostic difficulties clinicians face in assessing the course of severe COVID-19.

### COVID-19 with bacterial superinfection and antibiotic treatment

We report a rate of bacterial superinfection of 35.5% after tocilizumab treatment, which is associated with a higher mortality. This is within the range of reported superinfection rates: Yoon et al. report 30% of superinfections in patients with COVID-19 and intensive care unit (ICU) treatment [32], a Norwegian study showed a superinfection rate of 43% [33] in patients treated with dexamethasone. We observed a higher rate of antibiotic use compared to microbiological evidence of superinfection, consistent with studies demonstrating antibiotic overuse, partly attributed to the significant increase of inflammatory markers inherent to COVID-19 but often misinterpreted as evidence of bacterial superinfection [34]. A French study compared the risk of superinfection in patients treated with dexamethasone vs. dexamethasone plus tocilizumab treatment in an ICU setting and reported no difference in superinfection rates [35].

However, superinfection is an important complication of COVID-19 and the accuracy of bacterial infection diagnosis is critical for individual patient outcomes, but also for the control of antibiotic consumption and the incidence of infections by resistant pathogens [36]. This is even more true for patients receiving tocilizumab, being at an increased risk for severe and life-threatening infections. The knowledge of the trajectories and the predictive value of the inflammatory parameters we have described in this study may support clinicians in the early diagnosis and optimal treatment of secondary bacterial infections and thus contribute to the improvement of the clinical management of patients with severe COVID-19.

### Limitations

Our data are limited by their retrospective and single center nature. Most limiting is the fact that patients with different stages of COVID-19 are compared, especially since inflammatory markers vary at the various stages of COVID-19. We encourage the same analysis of IL-6 kinetics for larger cohorts of patients in the same stages of COVID-19.

## Supplementary Information

Below is the link to the electronic supplementary material.

Supplementary file1 (JPG 1336 KB)

## Data Availability

The underlying data are available upon reasonable request from the corresponding author.

## References

[1] Hu B, Huang S, Yin L. The cytokine storm and COVID-19. J Med Virol. 2021;93:250–6. 10.1002/jmv.26232. 32592501 10.1002/jmv.26232 PMC7361342

[2] Yuan Y, Jiao B, Qu L, Yang D, Liu R. The development of COVID-19 treatment. Front Immunol. 2023;14:1125246. 10.3389/fimmu.2023.1125246. 36776881 10.3389/fimmu.2023.1125246 PMC9909293

[3] Gottlieb RL, Vaca CE, Paredes R, Mera J, Webb BJ, Perez G, et al. Early remdesivir to prevent progression to severe Covid-19 in outpatients. N Engl J Med. 2022;386:305–15. 10.1056/NEJMoa2116846. 34937145 10.1056/NEJMoa2116846 PMC8757570

[4] Ganesh R, Philpot LM, Bierle DM, Anderson RJ, Arndt LL, Arndt RF, et al. Real-world clinical outcomes of Bamlanivimab and Casirivimab-Imdevimab among high-risk patients with mild to moderate coronavirus disease 2019. J Infect Dis. 2021;224:1278–86. 10.1093/infdis/jiab377. 34279629 10.1093/infdis/jiab377 PMC8344643

[5] Gupta A, Gonzalez-Rojas Y, Juarez E, Crespo Casal M, Moya J, Falci DR, et al. Early treatment for Covid-19 with SARS-CoV-2 neutralizing antibody sotrovimab. N Engl J Med. 2021;385:1941–50. 10.1056/NEJMoa2107934. 34706189 10.1056/NEJMoa2107934

[6] Hammond J, Leister-Tebbe H, Gardner A, Abreu P, Bao W, Wisemandle W, et al. Oral nirmatrelvir for high-risk, nonhospitalized adults with Covid-19. N Engl J Med. 2022;386:1397–408. 10.1056/NEJMoa2118542. 35172054 10.1056/NEJMoa2118542 PMC8908851

[7] Horby P, Lim WS, Emberson JR, Mafham M, Bell JL, Linsell L, et al. Dexamethasone in hospitalized patients with Covid-19. N Engl J Med. 2021;384:693–704. 10.1056/NEJMoa2021436. 32678530 10.1056/NEJMoa2021436 PMC7383595

[8] Scott LJ. Tocilizumab: a review in rheumatoid arthritis. Drugs. 2017;77:1865–79. 10.1007/s40265-017-0829-7. 29094311 10.1007/s40265-017-0829-7 PMC5736769

[9] Choy EH, de Benedetti F, Takeuchi T, Hashizume M, John MR, Kishimoto T. Translating IL-6 biology into effective treatments. Nat Rev Rheumatol. 2020;16:335–45. 10.1038/s41584-020-0419-z. 32327746 10.1038/s41584-020-0419-z PMC7178926

[10] Cortegiani A, Ippolito M, Greco M, Granone V, Protti A, Gregoretti C, et al. Rationale and evidence on the use of tocilizumab in COVID-19: a systematic review. Pulmonology. 2021;27:52–66. 10.1016/j.pulmoe.2020.07.003. 32713784 10.1016/j.pulmoe.2020.07.003 PMC7369580

[11] Hasanin A, Mostafa M. Tocilizumab in patients with COVID-19: which patient, time, and dose? J Anesth. 2021;35:896–902. 10.1007/s00540-021-02974-0. 34264384 10.1007/s00540-021-02974-0 PMC8280617

[12] Kow CS, Hasan SS. Interleukin-6 blockade with tocilizumab in COVID-19: does it live up to its hype? Pulmonology. 2021;27:86–7. 10.1016/j.pulmoe.2020.10.004. 33158786 10.1016/j.pulmoe.2020.10.004 PMC7577654

[13] Klopfenstein T, Gendrin V, Kadiane-Oussou NJ, Conrozier T, Zayet S. Tocilizumab in COVID-19 pneumonia: practical proposals based on a narrative review of randomised trials. Rev Med Virol. 2022;32:e2239. 10.1002/rmv.2239. 33882179 10.1002/rmv.2239 PMC8250236

[14] Bartoletti M, Azap O, Barac A, Bussini L, Ergonul O, Krause R, et al. ESCMID COVID-19 living guidelines: drug treatment and clinical management. Clin Microbiol Infect. 2022;28:222–38. 10.1016/j.cmi.2021.11.007. 34823008 10.1016/j.cmi.2021.11.007 PMC8606314

[15] Agarwal A, Hunt B, Stegemann M, Rochwerg B, Lamontagne F, Siemieniuk RA, et al. A living WHO guideline on drugs for covid-19. BMJ. 2020;370:m3379. 10.1136/bmj.m3379. 32887691 10.1136/bmj.m3379

[16] Nishimoto N, Terao K, Mima T, Nakahara H, Takagi N, Kakehi T. Mechanisms and pathologic significances in increase in serum interleukin-6 (IL-6) and soluble IL-6 receptor after administration of an anti-IL-6 receptor antibody, tocilizumab, in patients with rheumatoid arthritis and Castleman disease. Blood. 2008;112:3959–64. 10.1182/blood-2008-05-155846. 18784373 10.1182/blood-2008-05-155846

[17] Cremer S, Jakob C, Berkowitsch A, Borgmann S, Pilgram L, Tometten L, et al. Elevated markers of thrombo-inflammatory activation predict outcome in patients with cardiovascular comorbidities and COVID-19 disease: insights from the LEOSS registry. Clin Res Cardiol. 2021;110:1029–40. 10.1007/s00392-020-01769-9. 33211155 10.1007/s00392-020-01769-9 PMC7674577

[18] Freise NF, Gliga S, Fischer JC, Lübke N, Lutterbeck M, Schöler M, et al. Convalescent plasma treatment for SARS-CoV-2 infected high-risk patients: a matched pair analysis to the LEOSS cohort. Sci Rep. 2022;12:19035. 36351986 10.1038/s41598-022-23200-1 PMC9643921

[19] Gordon AC, Mouncey PR, Al-Beidh F, Rowan KM, Nichol AD, Arabi YM, et al. Interleukin-6 receptor antagonists in critically Ill patients with Covid-19. N Engl J Med. 2021;384:1491–502. 10.1056/NEJMoa2100433. 33631065 10.1056/NEJMoa2100433 PMC7953461

[20] Soin AS, Kumar K, Choudhary NS, Sharma P, Mehta Y, Kataria S, et al. Tocilizumab plus standard care versus standard care in patients in India with moderate to severe COVID-19-associated cytokine release syndrome (COVINTOC): an open-label, multicentre, randomised, controlled, phase 3 trial. Lancet Respir Med. 2021;9:511–21. 10.1016/S2213-2600(21)00081-3. 33676589 10.1016/S2213-2600(21)00081-3 PMC8078880

[21] Salvarani C, Dolci G, Massari M, Merlo DF, Cavuto S, Savoldi L, et al. Effect of tocilizumab vs standard care on clinical worsening in patients hospitalized with COVID-19 pneumonia: a randomized clinical trial. JAMA Intern Med. 2021;181:24–31. 10.1001/jamainternmed.2020.6615. 33080005 10.1001/jamainternmed.2020.6615 PMC7577199

[22] Stone JH, Frigault MJ, Serling-Boyd NJ, Fernandes AD, Harvey L, Foulkes AS, et al. Efficacy of tocilizumab in patients hospitalized with covid-19. N Engl J Med. 2020;383:2333–44. 10.1056/NEJMoa2028836. 33085857 10.1056/NEJMoa2028836 PMC7646626

[23] Coomes EA, Haghbayan H. Interleukin-6 in Covid-19: a systematic review and meta-analysis. Rev Med Virol. 2020;30:1–9. 10.1002/rmv.2141. 32845568 10.1002/rmv.2141 PMC7460877

[24] Verboogen DRJ, Revelo NH, Ter Beest M, van den Bogaart G. Interleukin-6 secretion is limited by self-signaling in endosomes. J Mol Cell Biol. 2019;11:144–57. 10.1093/jmcb/mjy038. 30016456 10.1093/jmcb/mjy038 PMC6392102

[25] Galván-Román JM, Rodríguez-García SC, Roy-Vallejo E, Marcos-Jiménez A, Sánchez-Alonso S, Fernández-Díaz C, et al. IL-6 serum levels predict severity and response to tocilizumab in COVID-19: an observational study. J Allergy Clin Immunol. 2021;147:72–80. 10.1016/j.jaci.2020.09.018. 33010257 10.1016/j.jaci.2020.09.018 PMC7525244

[26] Mandel M, Harari G, Gurevich M, Achiron A. Cytokine prediction of mortality in COVID19 patients. Cytokine. 2020;134:155190. 10.1016/j.cyto.2020.155190. 32673995 10.1016/j.cyto.2020.155190 PMC7351379

[27] Lakatos B, Szabo BG, Bobek I, Gopcsa L, Beko G, Kiss-Dala N, et al. Laboratory parameters predicting mortality of adult in-patients with COVID-19 associated cytokine release syndrome treated with high-dose tocilizumab. Acta Microbiol Immunol Hung. 2021. 10.1556/030.2021.01526. 34370690 10.1556/030.2021.01526

[28] Gager GM, Biesinger B, Hofer F, Winter M-P, Hengstenberg C, Jilma B, et al. Interleukin-6 level is a powerful predictor of long-term cardiovascular mortality in patients with acute coronary syndrome. Vascul Pharmacol. 2020;135:106806. 10.1016/j.vph.2020.106806. 33035661 10.1016/j.vph.2020.106806

[29] Pecoits-Filho R, Bárány P, Lindholm B, Heimbürger O, Stenvinkel P. Interleukin-6 is an independent predictor of mortality in patients starting dialysis treatment. Nephrol Dial Transplant. 2002;17:1684–8. 10.1093/ndt/17.9.1684. 12198224 10.1093/ndt/17.9.1684

[30] Berman M, Ben-Ami R, Berliner S, Anouk M, Kaufman I, Broyde A, et al. The effect of tocilizumab on inflammatory markers in patients hospitalized with serious infections. Case series and review of literature. Life (Basel). 2021. 10.3390/life11030258. 33804790 10.3390/life11030258 PMC8003879

[31] Hofmaenner DA, Wendel Garcia PD, Ganter CC, Brugger SD, Buehler PK, David S. What every intensivist should know about Tocilizumab. Crit Care. 2021;25:262. 10.1186/s13054-021-03696-1. 34315504 10.1186/s13054-021-03696-1 PMC8313874

[32] Yoon SM, Lee J, Lee S-M, Lee HY. Incidence and clinical outcomes of bacterial superinfections in critically ill patients with COVID-19. Front Med (Lausanne). 2023;10:1079721. 10.3389/fmed.2023.1079721. 36936237 10.3389/fmed.2023.1079721 PMC10017481

[33] Søvik S, Barrat-Due A, Kåsine T, Olasveengen T, Strand MW, Tveita AA, et al. Corticosteroids and superinfections in COVID-19 patients on invasive mechanical ventilation. J Infect. 2022;85:57–63. 10.1016/j.jinf.2022.05.015. 35605805 10.1016/j.jinf.2022.05.015 PMC9122884

[34] Granata G, Schiavone F, Pipitone G, Taglietti F, Petrosillo N. Antibiotics use in COVID-19 patients: a systematic literature review. J Clin Med. 2022. 10.3390/jcm11237207. 36498781 10.3390/jcm11237207 PMC9739751

[35] Camou F, Issa N, Hessamfar M, Guisset O, Mourissoux G, Pedeboscq S, et al. Is tocilizumab plus dexamethasone associated with superinfection in critically Ill COVID-19 patients? J Clin Med. 2022. 10.3390/jcm11195559. 36233432 10.3390/jcm11195559 PMC9573530

[36] Nedel W, da Silveira F, da Silva CF, Lisboa T. Bacterial infection in coronavirus disease 2019 patients: co-infection, super-infection and how it impacts on antimicrobial use. Curr Opin Crit Care. 2022;28:463–9. 10.1097/MCC.0000000000000975. 36017559 10.1097/MCC.0000000000000975 PMC9593329

