# Supplementary figures and images for: Trajectories and predictive significance of inflammatory parameters for clinical outcome in COVID–19 patients treated with tocilizumab
Source: Infection. 2024 Aug 29;53(1):339–48. doi: 10.1007/s15010-024-02375-x (PMC11825610; doi:10.1007/s15010-024-02375-x)

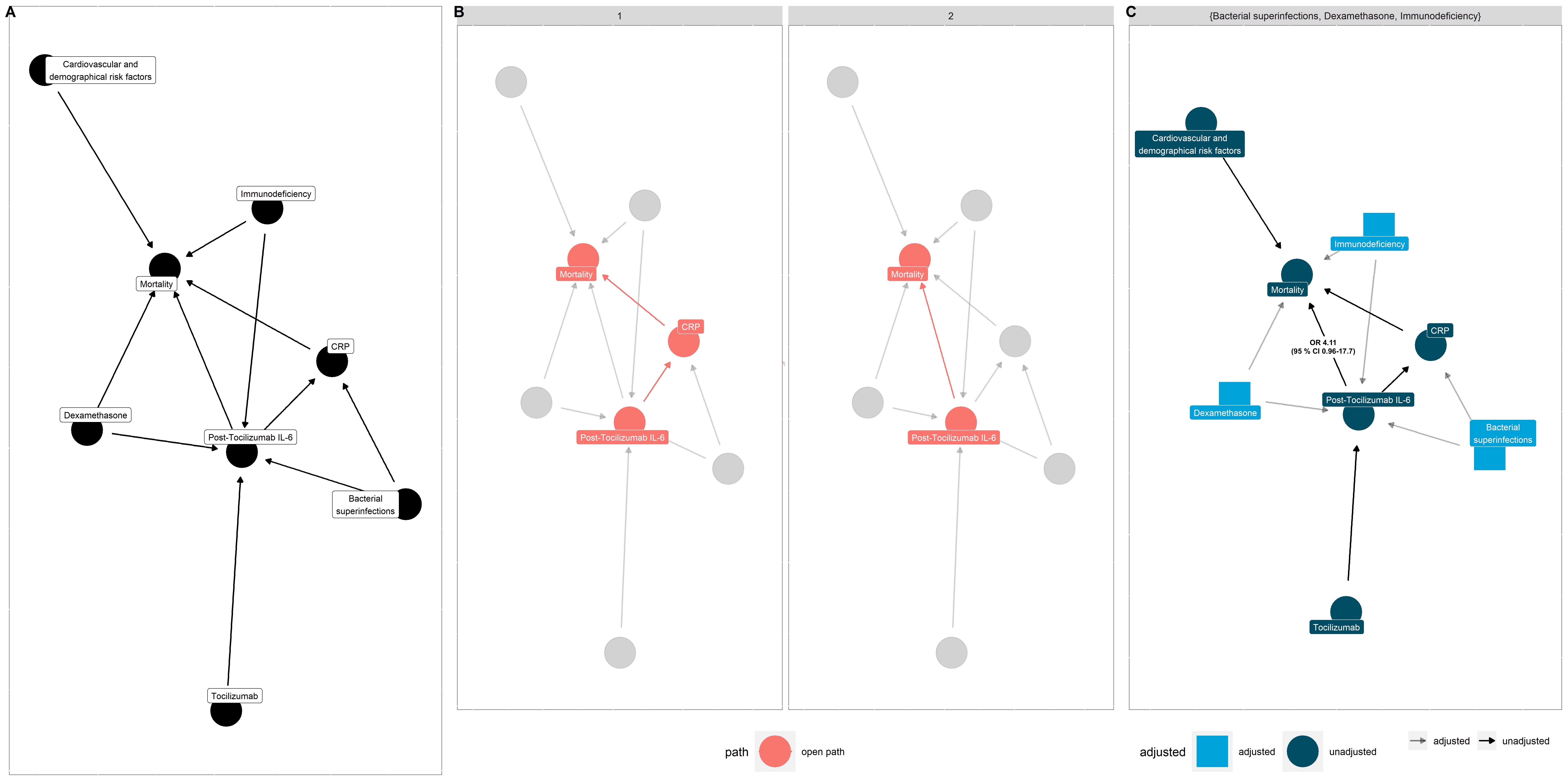

Supplement: Supplementary file 1 — Supplementary file1 (JPG 1336 KB) [file 15010_2024_2375_MOESM1_ESM.jpg]
